# Designing a nitrogen-efficient cold-tolerant maize for modern agricultural systems

**DOI:** 10.1093/plcell/koaf139

**Published:** 2025-07-17

**Authors:** Jonathan Odilón Ojeda-Rivera, Allison C Barnes, Elizabeth A Ainsworth, Ruthie Angelovici, Bruno Basso, Lara J Brindisi, Matthew D Brooks, Wolfgang Busch, Gretta L Buttelmann, Michael J Castellano, Junping Chen, Denise E Costich, Natalia de Leon, Bryan D Emmett, David Ertl, Sarah L Fitzsimmons, Sherry A Flint-Garcia, Michael A Gore, Kaiyu Guan, Charles O Hale, Sam Herr, Candice N Hirsch, David H Holding, James B Holland, Sheng-Kai Hsu, Jian Hua, Matthew B Hufford, Shawn M Kaeppler, Emma N Leary, Zong-Yan Liu, Anthony A Mahama, Tyler J McCubbin, Carlos D Messina, Todd P Michael, Sara J Miller, Seth C Murray, Sakiko Okumoto, Elad Oren, Alexa N Park, Miguel A Piñeros, Nicholas Ace Pugh, Victor Raboy, Rubén Rellán-Álvarez, M Cinta Romay, Travis Rooney, Rebecca L Roston, Ruairidh J H Sawers, James C Schnable, Aimee J Schulz, M Paul Scott, Nathan M Springer, Jacob D Washburn, Michelle A Zambrano, Jingjing Zhai, Jitao Zou, Edward S Buckler

**Affiliations:** Institute for Genomic Diversity, Cornell University, Ithaca, NY 14853, USA; USDA-ARS Plant Science Research Unit, Raleigh, NC 27606, USA; Department of Plant Biology, University of Illinois Urbana-Champaign, Urbana, IL 61801, USA; Department of Crop Sciences, University of Illinois Urbana-Champaign, Urbana, IL 61801, USA; Division of Biological Sciences, Interdisciplinary Plant Group, Christopher S. Bond Life Sciences Center, University of Missouri, Columbia, MO 65211, USA; Department of Earth and Environmental Sciences and W.K. Kellogg Biological Station, Michigan State University, East Lansing, MI 48823, USA; USDA-ARS Plant, Soil and Nutrition Research, Ithaca, NY 14853, USA; USDA-ARS Global Change and Photosynthesis Research Unit, Urbana, IL 61801, USA; Plant Molecular and Cellular Biology Laboratory, The Salk Institute for Biological Studies, La Jolla, CA 92037-100210, USA; Department of Ecology, Evolution, and Organismal Biology, Iowa State University, Ames, IA 50011, USA; Department of Agronomy, Iowa State University, Ames, IA 50011, USA; USDA-ARS, Cropping Systems Research Laboratory, Plant Stress and Germplasm Development Unit, Lubbock, TX 79415, USA; Institute for Genomic Diversity, Cornell University, Ithaca, NY 14853, USA; Department of Plant and Agroecosystem Sciences, University of Wisconsin, Madison, WI 53706, USA; USDA-ARS, National Laboratory for Agriculture and the Environment, Ames, IA 50011, USA; Iowa Corn Promotion Board (Retired), Johnston, IA 50131, USA; Division of Biological Sciences, University of Missouri, Columbia, MO 65211, USA; USDA-ARS Plant Genetics Research Unit, Columbia, MO 65211, USA; Plant Breeding and Genetics Section, School of Integrative Plant Science, Cornell University, Ithaca, NY 14853, USA; Agreocosystem Sustainability Center and Department of Natural Resources and Environmental Sciences, University of Illinois Urbana-Champaign, Urbana, IL 61801, USA; Plant Breeding and Genetics Section, School of Integrative Plant Science, Cornell University, Ithaca, NY 14853, USA; Plant Breeding and Genetics Section, School of Integrative Plant Science, Cornell University, Ithaca, NY 14853, USA; Department of Agronomy and Plant Genetics, University of Minnesota, St Paul, MN 55108, USA; Department of Agronomy and Horticulture and Center for Plant Science Innovation, University of Nebraska-Lincoln, Lincoln, NE 68588, USA; USDA-ARS Plant Science Research Unit, Raleigh, NC 27606, USA; Institute for Genomic Diversity, Cornell University, Ithaca, NY 14853, USA; Plant Biology Section, School of Integrative Plant Science, Cornell University, Ithaca, NY 14853, USA; Department of Ecology, Evolution, and Organismal Biology, Iowa State University, Ames, IA 50011, USA; Department of Plant and Agroecosystem Sciences, University of Wisconsin, Madison, WI 53706, USA; Wisconsin Crop Innovation Center, Middleton, WI 53562, USA; Division of Plant Science and Technology, University of Missouri, Columbia, MO 65211, USA; Plant Breeding and Genetics Section, School of Integrative Plant Science, Cornell University, Ithaca, NY 14853, USA; Department of Agronomy, Iowa State University, Ames, IA 50011, USA; USDA-ARS Plant Genetics Research Unit, Columbia, MO 65211, USA; Department of Horticultural Sciences, University of Florida, Gainesville, FL 32611, USA; Plant Molecular and Cellular Biology Laboratory, The Salk Institute for Biological Studies, La Jolla, CA 92037-100210, USA; Institute for Genomic Diversity, Cornell University, Ithaca, NY 14853, USA; Department of Soil and Crop Sciences, Texas A&M University, College Station, TX 77843, USA; Department of Soil and Crop Sciences, Texas A&M University, College Station, TX 77843, USA; Newe Ya’ar Research Center, Institute of Plant Sciences, Agricultural Research Organization, Volcani Institute, Ramat Yishay 3009500, Israel; Department of Soil and Crop Sciences, Texas A&M University, College Station, TX 77843, USA; USDA-ARS Robert W. Holley Center for Agriculture and Health, Ithaca, NY 14853, USA; USDA-ARS, Cropping Systems Research Laboratory, Plant Stress and Germplasm Development Unit, Lubbock, TX 79415, USA; Independent Researcher, Portland, OR, USA; Department of Molecular and Structural Biochemistry, N.C. Plant Sciences Initiative, North Carolina State University, Raleigh, NC 27695, USA; Institute for Genomic Diversity, Cornell University, Ithaca, NY 14853, USA; Sesaco Corporation, Austin, TX 78701, USA; Department of Biochemistry, Center for Plant Science Innovation, University of Nebraska-Lincoln, Lincoln, NE 68503, USA; Department of Plant Science, The Pennsylvania State University, State College, PA 16802, USA; Department of Agronomy and Horticulture and Center for Plant Science Innovation, University of Nebraska-Lincoln, Lincoln, NE 68588, USA; Plant Breeding and Genetics Section, School of Integrative Plant Science, Cornell University, Ithaca, NY 14853, USA; Department of Agronomy, Iowa State University, Ames, IA 50011, USA; Department of Plant and Microbial Biology, University of Minnesota, St. Paul, MN 55108, USA; USDA-ARS Plant Genetics Research Unit, Columbia, MO 65211, USA; Department of Plant Biology, University of Illinois Urbana-Champaign, Urbana, IL 61801, USA; Department of Crop Sciences, University of Illinois Urbana-Champaign, Urbana, IL 61801, USA; Institute for Genomic Diversity, Cornell University, Ithaca, NY 14853, USA; Department of Plant Science, The Pennsylvania State University, University Park, PA 16802, USA; Institute for Genomic Diversity, Cornell University, Ithaca, NY 14853, USA; Plant Breeding and Genetics Section, School of Integrative Plant Science, Cornell University, Ithaca, NY 14853, USA; USDA-ARS Robert W. Holley Center for Agriculture and Health, Ithaca, NY 14853, USA

## Abstract

Maize (*Zea mays* L.) is the world's most productive grain crop and a cornerstone of global food supply. However, in temperate agricultural systems, maize exhibits 2 key anomalies. First, as a tropical species, maize cannot be planted in the cold conditions of early spring when light and natural soil nitrogen are available, resulting in a shorter growing season and creating a seasonal mismatch between nitrogen accessibility and demand. Second, maize kernel protein is a major nitrogen sink, driving fertilizer demand because of the scale of cultivation. This inefficient mismatch stems from modern maize's uses and the modest nutritional value of storage proteins. To address these anomalies, we established the Circular Economy that Reimagines Corn Agriculture initiative. Our vision requires advances in 3 research areas: (ⅰ) developing cold and frost tolerance during germination and early growth to enable the use of spring nitrogen and light resources; (ⅱ) reducing nitrogen allocation to grain by reducing low-quality storage proteins and developing alternative nitrogen sinks; and (ⅲ) stabilizing soil nitrogen by enhancing biological nitrification inhibition. We present blueprints for a nitrogen-efficient, cold-tolerant maize designed to utilize the full growing season, enabling farmers in temperate regions to fully leverage maize's C4 photosynthesis, reduce fertilizer inputs, increase yields, and minimize environmental impact.

## Introduction

Agriculture occupies nearly 50% of the Earth's land (Ritchie and Roser 2019), yet maize, cultivated on just 1.3% of this area, produces a caloric output equivalent to what half of the global population consumes in a year (Erenstein et al. 2022). Over 60% of global maize production takes place in 3 industrialized temperate agricultural regions: the United States of America (US), China, and the European Union (Erenstein et al. 2022; FAOSTAT 2024). In these regions, maize serves as the dominant source of calories for livestock, providing 30% to 85% for cattle, poultry, and swine (FAOSTAT 2024). It is also key for starch, sugar, and biofuel production. While an incredibly successful crop, its evolutionary origins are still limiting its maximal efficiency in these modern agricultural systems.

While maize is very high-yielding and land-efficient, 50% to 60% of US nitrogen fertilizer supports a grain crop that produces 9 times more starch than protein (Zhang et al. 2015a, 2021; Yu and Moon 2021). Although nitrogen is essential for powering photosynthesis and driving high yields (Cao et al. 2017), the photosynthetic canopy of maize operates at full efficiency for only about a quarter of the temperate year. As a result, much of the applied nitrogen is susceptible to loss, both on the farm and further downstream. Only 11% of the nitrogen applied in US crop production ultimately reaches human consumption (Sabo et al. 2019). These nitrogen losses cost farmers over $10 billion annually in fertilizer expenses (Gillespie 2025), are responsible for over 70% of water pollution (Alexander et al. 2008), and contribute 5% of total greenhouse gas emissions through N_2_O emissions (EPA 2022). The inefficiencies in maize production can be traced back to its evolutionary history, domestication, tropical origin, and grain composition.

The history starts over 20 million years ago when decreasing atmospheric CO_2_—still about 50% higher than today (Cenozoic CO2 Proxy Integration Project Consortium 2023)—and increasing aridity drove the evolution of maize's ancestors, the Andropogoneae grasses (Bianconi et al. 2020). These environmental pressures led to the development of C4 photosynthesis, specifically the NADP-ME subtype, a remarkable adaptation that functions efficiently under lower CO_2_ levels and high-temperature conditions (Sage et al. 2012). By reducing photorespiration, NADP-ME C4 photosynthesis enabled higher photosynthetic rates while minimizing nitrogen investment in enzymes, thereby significantly improving nitrogen- and water-use efficiency (Hatch 1987; Sage and Monson 1998). Specifically, the Andropogoneae tribe, which utilizes the NADP-ME subtype, exhibits photosynthetic nitrogen-use efficiency that is approximately twice as high as that of C3 plants and about 20% greater than other C4 subtypes (e.g. NAD-ME and PEPCK; Ghannoum et al. 2005; Wang et al. 2014; Bianconi et al. 2020).

**Figure 1.**
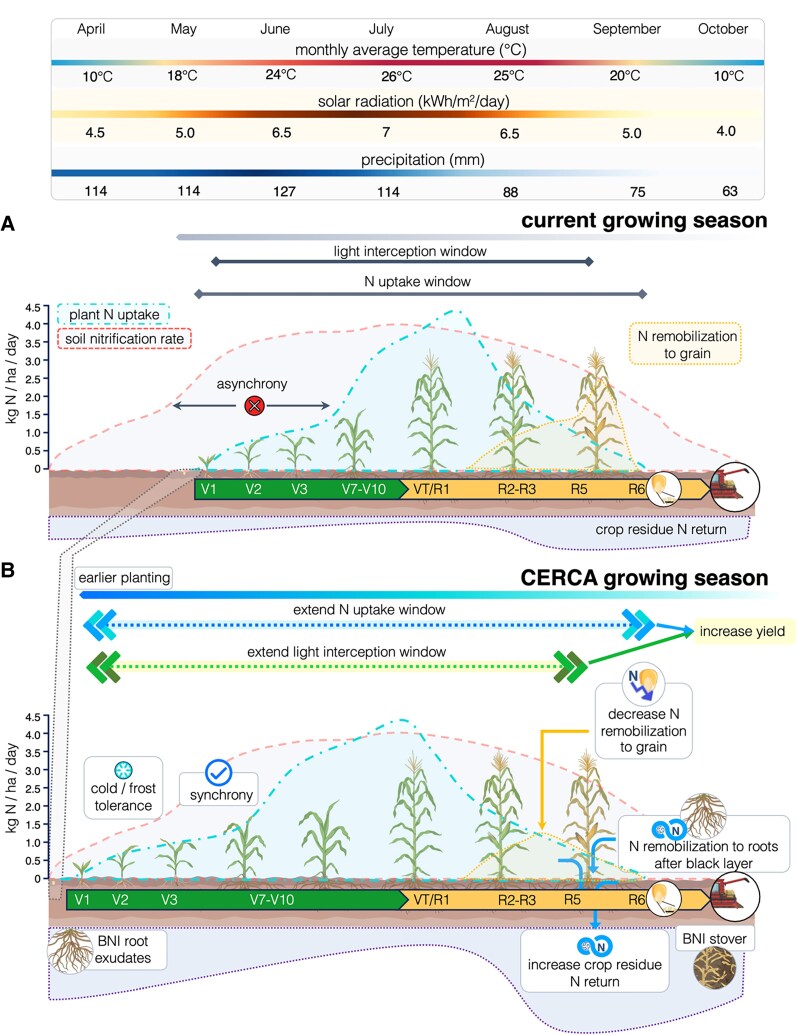
Seasonal patterns in current temperate maize agriculture and the proposed CERCA ideotype. **A)** Current growing season in the US Midwest. Average temperature and precipitation data in Iowa from 1991 to 2020 (NCEI 2021); Average solar irradiation in Iowa from 2014 to 2023 (NREL 2024). Maize nitrogen uptake was based on Bender et al. (2013). Developmental stages (V, Vegetative [V1-10]; VT, Vegetative Tasseling [VT]; R, Reproductive [R1–R6]) are based on Abendroth et al. (2011). **B)** Proposed extended growing season enabled by the CERCA ideotype. Panel portrays an expanded growing season facilitated by CERCA traits. By integrating these traits, maize can leverage an extended window for light interception and nitrogen synchronization, enabling higher carbohydrate yields, reduced nitrogen losses, and improved sustainability. The CERCA traits depicted here are explained throughout the text. BNI, Biological Nitrification Inhibition.

These evolutionary breakthroughs enabled Andropogoneae grasses to dominate 17% of the planet's vegetation, supporting some of the largest herds of hoofed animals (Welker et al. 2020). Later, approximately 6,000 to 10,000 years ago, maize, sorghum, and sugarcane were domesticated from the Andropogoneae in the tropical regions of Mexico, northeastern Africa, and New Guinea, respectively (Dillon et al. 2007; Stitzer and Ross-Ibarra 2018). In the tropics, grasses have a longer growing season, but less soil nutrients, while in temperate grasslands it is frequently the reverse (Hernández-Romero et al. 2024). Maize is not cold-tolerant and thus, cannot have a long growing season in temperate regions (Fig. 1A). The shorter growing season limits the ability to fully exploit maize's highly efficient C4 photosynthesis, by effectively reducing the time available to fix carbon and accumulate biomass.

The shorter season also results in a mismatch between the soil microbe activities (usually active in early spring, before corn is planted) that catalyze nitrogen cycling and plant growth (Wagner-Riddle et al. 2017; Lawrence et al. 2021). This asynchrony is particularly problematic in temperate regions, like the US Midwest, and results in peak nitrogen losses to the environment in spring (Wagner-Riddle et al. 2017; Lawrence et al. 2021; Fig. 1A). Winter crops with split fertilizer application can reduce spring losses (Gentry et al. 2024). Similarly, an earlier maize planting could allow for a powerful uptake by mid-May and all of June, synchronizing crop nitrogen uptake with soil nitrogen cycling (Fig. 1B).

**Figure 2.**
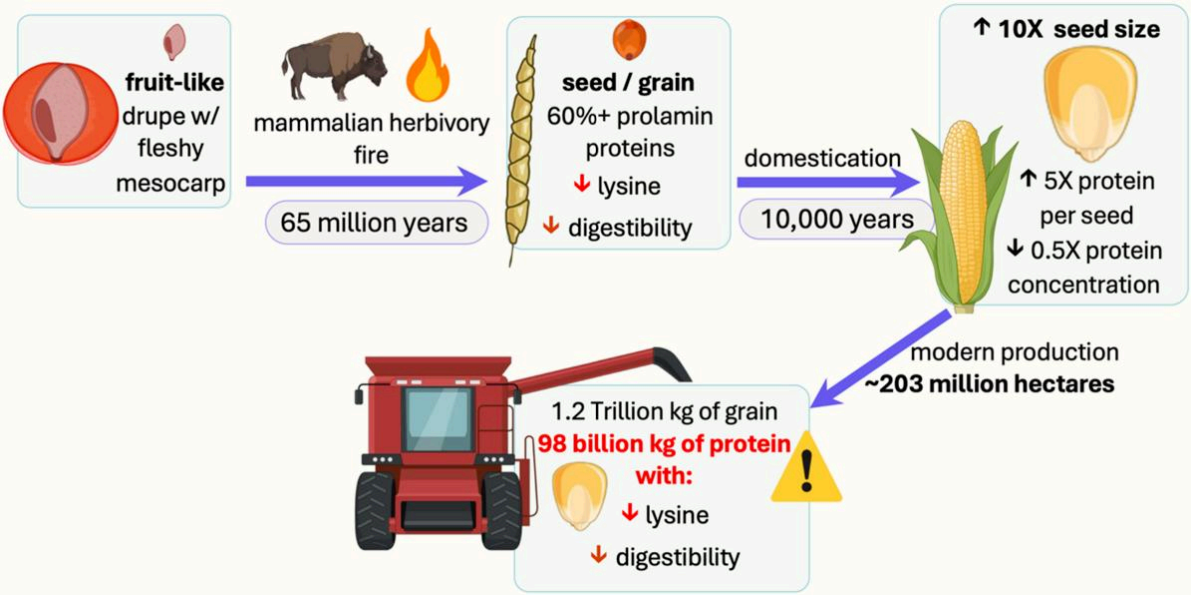
Maize's evolutionary history and its relationship with the production of protein with marginal value.

Beyond limiting its growing season, evolutionary and domestication history has also shaped how maize allocates nitrogen to its seed, further contributing to inefficiencies in downstream grain processing (Fig. 2). Grass ancestors likely had a fleshy fruit (drupe; Kellogg 2000; Rudall et al. 2005). Grass species then evolved unique seeds with grain proteins of poor nutritional quality (low lysine content, insoluble, and less digestible) as the result of millions of years of fire ecology and herbivory pressure, where the herbivore targets the leaf for nutrition and the seed is passed through the gut and dispersed (Janzen 1984). Moreover, domestication favored even larger seeds (Liu et al. 2016). The result is maize having 5 times more total protein per seed than its wild relative teosinte, which already had large seeds (Fig. 2; Flint-Garcia et al. 2009). Conserving and passing on nitrogen to the next generation makes evolutionary sense for an annual species like maize. But it is a poor fit for a species in an agricultural system where over 90% of its downstream uses value the starch, not the proteins (Yu and Moon 2021; Zhang et al. 2021). Today, this nitrogen would be better used to extend photosynthesis into the fall and then be recycled on farm for next season. Domesticated crops like sugarcane, sorghum, and maize were cultivated for storable carbohydrates rather than their proteins, which were obtained through animal hunting and legumes. Industrialized maize agriculture in temperate environments differs greatly from these early practices in many areas, yet 1 fundamental goal role remains the same: convert as much sunlight and CO₂ into storable carbohydrates as possible.

In summary, we can identify 2 anomalies in the current temperate corn cropping system.

1. There is a seasonal mismatch between nitrogen and light availability. As a tropical species, maize cannot be planted in early spring, which shortens maize's photosynthetic window and results in the microbial production of nitrogen from soil organic matter exceeding crop demand for a long portion of the growing season (Rogovska et al. 2023).
2. Maize grain contains an excess of storage proteins with marginal food system value that drive unnecessary nitrogen demand due to the massive scale of corn production. Given the majority of downstream uses of maize (livestock feed and industrial applications), maize is primarily valued for its high-quality storable starch, yet its grain still contains 8% to 10% protein (Hasjim et al. 2009; Loy and Lundy 2019) and 60% of the applied nitrogen (Zhang et al. 2015a). Given the scale of maize production (Fig. 2), every protein percentage change has relatively large repercussions on fertilizer inputs and downstream markets.

To address these anomalies, we have established the Circular Economy that Reimagines Corn Agriculture (CERCA) initiative that aims to solve them through a combination of multidisciplinary approaches including modeling, genetics, physiology, and agronomy. These maize agriculture anomalies are, in fact, opportunities to design a corn crop that is more efficient and that reduces costs and externalities to farmers and society at large.

### The CERCA ideotype: goals, benefits, traits, and feasibility

The breeding concept of our initiative boosts starch and carbon production per unit area while synchronizing growth with the natural nitrogen cycle to maximize nitrogen efficiency and reduce environmental impact. The CERCA ideotype focuses on 3 key research areas: (ⅰ) improving seedling cold tolerance to enable earlier planting and extend the growing season, (ⅱ) recycling nitrogen on farm by reducing grain nitrogen demand and activating late-season nitrogen sinks, and (ⅲ) stabilizing nitrogen on farm by promoting biological nitrification inhibition (BNI). In the following, we outline specific goals, potential benefits, required traits, and feasibility. To provide context, we also address some frequently asked questions (FAQs), such as why CERCA focuses on novel approaches rather than improving standard agronomic nitrogen use efficiency (NUE) alone, and how these innovations can optimize nitrogen cycling across scales from farm-level efficiency to broader impacts on the food production system.

1. Improve seedling cold tolerance to enable earlier planting and extend the growing season. Goal: Shift average planting dates from May to early April in the US Corn Belt. Benefit: Corn yields could increase by over 10% due to earlier canopy closure before the summer solstice, and nitrogen losses could decrease by 20% or more (Fig. 1B). Inspired by the yield difference between winter and spring wheat (Entz and Fowler 1991; Fowler 2012), in warmer regions, winter annual planting could bring even larger gains if frost tolerance is improved to −5 °C (Gulf Coast) or −10 °C (greater Southeastern US). Currently, planting windows are already risky due to wet field conditions and equipment demands. Delaying plating shortens the growing season and increases heat and drought exposure. Expanding the planting window reduces these risks, but cooler soil and air temperatures, and frost risk, create new challenges. Traits: For April 1st planting in Iowa to be viable, maize germination would need to begin at 6 °C instead of the current 10 °C. Leaf emergence should follow a few weeks later when average high temperatures reach 18 °C, frost events are rare (with leaves tolerating temperatures as low as −2 °C), and sunny days maximize growth and nitrogen uptake. Trait Feasibility: We believe it is possible to improve frost tolerance in maize, because the capacity for this trait is represented in other closely related taxa (Hope and McElroy 1990; Naidu et al. 2003; Yan et al. 2019). Other grasses like wheat and rye germinate at nearly freezing temperatures and have much lower base temperatures for growth (Porter and Gawith 1999). For example, Tripsacum (a tropical and temperate sister genus) with stand frosts as low as −6 °C, and *Miscanthus* (a warm-season Andropogoneae with enhanced cold tolerance) photosynthesizes well in the US Corn Belt spring (deWet et al. 1982; Dohleman and Long 2009; Yan et al. 2019).
2. *On farm nitrogen recycling*: Goal: Reduce field nitrogen removal by 50% and keep nitrogen in a reusable labile form on farm for the next season. Benefit: This could reduce nitrogen fertilizer demand by up to 50% and especially decrease the need to apply nitrogen at the times when nitrogen losses are most likely. Grain with high starch is valuable to most markets (Yu and Moon 2021; Zhang et al. 2021), while reductions in low-quality storage proteins can be complemented by alternate nitrogen feeds for livestock (Sajeev et al. 2018; Cappelaere et al. 2021). High nitrogen in leaves through grain fill, supported by the stay-green trait (Thomas and Ougham 2014), can increase starch yields. Traits: Reduce grain nitrogen by 50% through a reduction of storage proteins and recycle leaf nitrogen at the end of season to root, stalk, and/or cob sinks. Trait Feasibility: The high levels of low-quality storage protein per seed is a byproduct of domestication for large seeds (Liu et al. 2016) and can be reduced as shown by selection experiments (Dudley and Lambert 2010) without compromising yield (Woore et al. 2021). Reducing grain nitrogen content is feasible as it is negatively or poorly correlated with yield in corn (Tenorio et al. 2019). Although modern hybrids exhibit an increasing yield response to nitrogen inputs (Haegele et al. 2013; Mueller et al. 2019a, 2019b), yield gains under high nitrogen conditions have occurred in parallel to a decline in grain protein content (Haegele et al. 2013). Likewise, historical data from commercial hybrids show a consistent decline in grain nitrogen content over the past decades (1946 to 2015), even as yields have continued to rise (DeBruin et al. 2017; Mueller et al. 2019a, 2019b). Grain nitrogen is primarily used for germination (Holding and Larkins 2008), but this is less of a concern in hybrid seed systems where replanting is unnecessary. However, potential impacts on germination should still be monitored.

Lower grain nitrogen also enables delayed leaf senescence and maintenance of photosynthetic proteins through grain fill; this functional stay-green genetics already exists in elite maize germplasm and transgenes show it can contribute to high yield (Thomas and Ougham 2014; Mueller et al. 2019a; Zhang et al. 2019). Indeed, selection for yield leads to a correlated retention of nitrogen in leaves at the expense of shoots and a reduction of grain nitrogen (Mueller et al. 2019a). Data from wheat indicates that reducing nitrogen content in grain can actually improve nitrogen utilization efficiency (Barraclough et al. 2010). Remobilization of nitrogen to storage organs and roots is found in perennial relatives of maize (Heggenstaller et al. 2009; Kiniry et al. 2024) and other members of the Andropogoneae tribe that remobilize up to 60% of the nitrogen present in aboveground tissues to underground tissues at the end of the season (Yang et al. 2009, 2016; Mitros et al. 2020; Li et al. 2024).

3. *Stabilizing nitrogen on farm by promoting BNI:* Goal: Maintain nitrogen in stable forms over winter and spring using BNI. Benefit: BNI has the potential to reduce nitrogen losses and increase plant-available nitrogen. Traits: BNI exudates have shown promise in tropical wheat (Subbarao et al. 2021) and perennial *Brachiaria humidicola* (Subbarao et al. 2009). Currently, the efficacy of BNI in temperate maize is challenging because of low root biomass during peak nitrification. However, root exudate-mediated BNI may prove helpful in the ideotype of earlier planting and improved synchrony of root growth with soil nitrogen mineralization and nitrification. Additionally, under the scenario of nitrogen recycling, BNI compounds exuded from roots or released upon residue decomposition could help immobilize nitrogen until uptake. Trait Feasibility: BNI compounds are produced by maize (Otaka et al. 2022, 2023) and likely serve additional defense roles. However, effective concentrations, residence time, and microbial interactions influencing the efficacy of these compounds remain unknown. End-of-season BNI compound accumulation from degrading crop residues (stalks, leaves, and roots that are magnitudes larger in mass than exudates) could remain active in early spring before planting, amplifying benefits. In fact, stover removal increases spring N_2_O emissions by up to 95% (Drury et al. 2021). Beyond maize BNI, winter cash or cover crops may be necessary to retain recycled nitrogen on farm. Targeted BNI development within these species may further enhance nitrogen retention.

### CERCA FAQs

**Figure 3.**
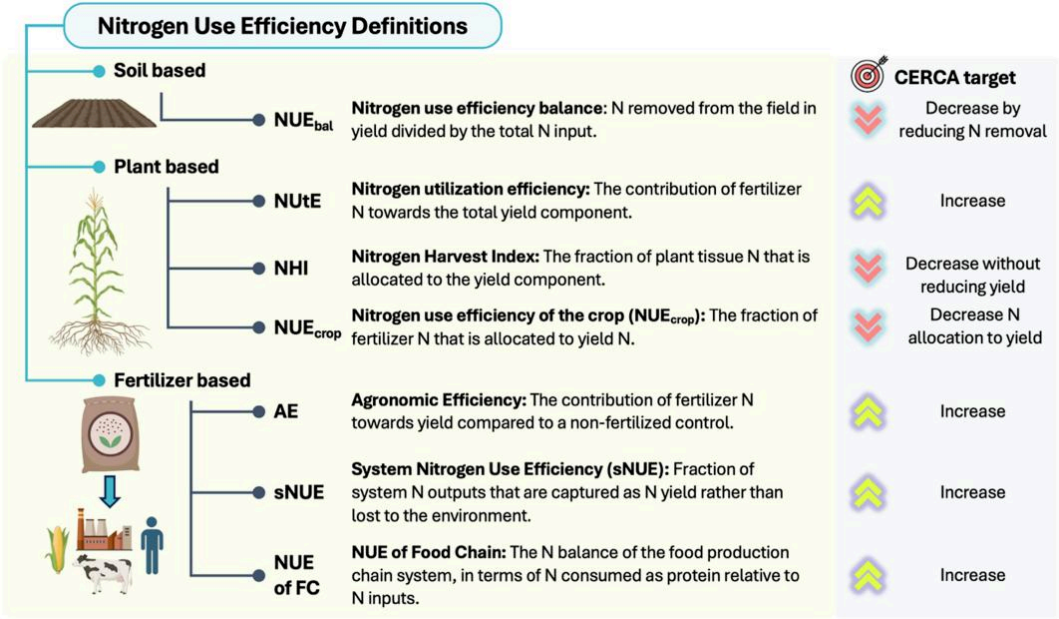
Definitions of NUE. NUE is a broad concept with different meanings, throughout this work we use multiple NUE definitions from (Congreves et al. 2021). Here we highlight the key definitions of NUE for CERCA and our targets.

- *Why not focus on standard NUE of the crop?* NUE is a broad concept with different formulations depending on the nitrogen sources contributing to crop production, as well as the interplay between soil, plant, and management practices (See Fig. 3 for targets and specific definitions of NUE; Congreves et al. 2021). Seed companies have already accomplished massive gains in the NUE of maize (Mueller et al. 2019a) with great genetics and physiology for acquiring nitrogen in the middle of the season. There are certainly more advances to be made but likely focusing on early and late season nitrogen use. CERCA aims to optimize *system-wide* NUE and the *overall* NUE of the food production chain by decreasing the amount of nitrogen removed from the farm while maintaining or reducing fertilizer application while also maintaining or increasing yield.
- *Why not focus on diversification of the system?* There are many reasons cropping systems should be diversified beyond the corn-soybean rotations common in the US Corn Belt. We believe there are tremendous opportunities for integrating a wide range of winter crops including wheat, biofuel canola, and legumes into a corn rotation system that could combine well with addressing food, fuel, and protein markets and enhance system level balance. Yet, there are many socio-techno-political factors outside of science that constrain farmer decisions, including equipment investments, farmer experience, processing infrastructure, effective transgenic trait packages, insurance, market structure, and efficiency. This, coupled with the lack of genotypes adapted to the Midwest and can be grown in double cropping systems, makes diversification of the system infeasible. Given the variety of complicated, intractable factors driving the US corn-soy agricultural system, transformational research is likely to involve approaches that evolve the crops and systems we use today, at least for the foreseeable future.
- *Why not focus on nitrogen-fixing maize?* First, we think it is tenable to make a nitrogen fixing maize variety of value in regions without synthetic nitrogen sources. However, nitrogen fixation is most likely to provide more nitrogen mid-season during grain fill (in soybean this occurs at R3 to R5; Córdova et al. 2019), when maize least needs it (Osterholz et al. 2017). Second, nitrogen symbiosis is metabolically expensive (Holland et al. 2023). Third, maize protein has low market value (Yu and Moon 2021; Zhang et al. 2021). Fourth, maize's land-use is massive (Cao et al. 2017; Erenstein et al. 2022); any reduction in starch yield due to rerouting energy for nitrogen fixation might result in increased land-use. In contrast, synthetic nitrogen fixation is highly land-use efficient as it does not require extensive cropland or biomass feedstocks, relying instead on industrial processes like the Haber-Bosch method to convert atmospheric nitrogen into ammonia-based fertilizers (Erisman et al. 2008). Synthetic nitrogen production is poised to become one of the first major consumers of renewable hydrogen, which can replace fossil-derived hydrogen in ammonia synthesis, significantly reducing off-target gas emissions from fertilizer manufacturing (IEA—Ammonia Technology Roadmap 2021).
- *What about phosphorus?* Phosphorus, an expensive and often imported farm input, shows similar inefficiencies as nitrogen. Too much phosphorus is translocated to the seed and stored in forms that are antinutritional to animals (Raboy 2020), similar to the evolutionary pressures shaping storage proteins. Reducing grain phosphorus can be an effective solution to increase phosphorus use efficiency (Rose et al. 2013) and aligns well with the CERCA objective to reduce grain nitrogen. Phytic acid locks phosphorus in the seed. Thus, reducing phytic acid levels has been a target to increase digestibility by monogastric animals (Dersjant-Li et al. 2015), to make other micronutrients more available, and to reduce phosphorus waste (Raboy 2001). Strategies to decrease phosphorus allocation to the grain include decreasing phytate synthesis and translocation to the grain (Raboy 2009, 2020; Ojeda-Rivera et al. 2022). Decreasing phytate content through the knocking-out of a grain transporter protein has been shown to be successful in rice (Yamaji et al. 2017) and barley (Bregitzer et al. 2007), demonstrating this approach is promising in grasses. Transgenic expression of phytases in the seed has also been successful and has been deregulated by APHIS. Moreover, phosphorus and nitrogen physiologies and signaling pathways are related to each other (Medici et al. 2015; Torres-Rodríguez et al. 2021), particularly during grain filling (Peng et al. 2024). This should be considered for nitrogen and phosphorus targets to avoid detrimental effects (Raboy et al. 1989) and to harness shared signaling pathways to reduce both nitrogen and phosphorus content in seed.
- *Why not perennial maize?* Like nitrogen fixation, perennializing maize is genetically possible, given maize's wild perennial relatives. However, physiological modeling and the current distribution of crops suggest there are strong tradeoffs between perennial root mass and seed yield (Vico and Brunsell 2018). In natural systems, perennials are most effective at producing biomass, not seeds (Hjertaas et al. 2022). Where water is available, perennial relatives like *Miscanthus* can fix more carbon per year than annual maize because of their extended growing season (Dohleman and Long 2009), compensating for the maintenance of extensive roots, which are energetically expensive. However, the time required to domesticate and breed a perennial maize, the disease pressure from a lack of rotation, the capital investment of changing equipment, and necessity of growers agreeing to adopt it, makes the implementation of perennial maize socio-techno-politically challenging. Instead, perennials could play a key role in precision conservation and biomass production. About 20% of US corn/soy acreage is not profitable (Basso et al. 2019; Basso and Antle 2020; Basso 2021). Perennial biomass crops could be used in these areas, where species like *Miscanthus*, switchgrass or other native perennials would produce biomass well, sequester soil carbon, and reduce N2O emissions to the atmosphere and nitrate leaching to groundwater. Finally, while CERCA is not aiming to develop a perennial maize, the traits being sourced from perennials—such as cold tolerance and nutrient remobilization—would be essential for such a crop. Over time, perennial maize might prove to be the most suitable option for certain environments and farming systems.
- *What about agronomic management practices to improve cold tolerance?* While the focus of our initiative is primarily on genetic and physiological interventions, we recognize there are several agronomic practices that can enhance cold tolerance, particularly during early development. These include mulching to regulate soil temperature (Bu et al. 2013), silicon application to promote root growth under cold stress (Moradtalab et al. 2018), biofertilizers and plant-growth-promoting microorganisms to stimulate development under low-temperature conditions (Li et al. 2020; Zhang et al. 2023), and temperature-activated seed coatings that delay germination until conditions are favorable (Archer and Gesch 2003; Pedrini et al. 2017). These approaches are not incompatible with the CERCA ideotype and could offer synergistic benefits in the field when aligned with the right genotype and environmental context.

## Engineering the CERCA ideotype: molecular, physiological, and genetic basis

Maize breeding in temperate regions is highly advanced, producing thousands of hybrids optimized for diverse environments and geographies. Selection can eventually produce hybrids with some of the characteristics of the CERCA ideotype; however, deep insight into physiological mechanisms, gene networks, and natural variants that drive key traits can hasten genetic gains. Time is of the essence when we seek a large reduction in nitrogen losses to the environment. To have an impact on maize breeding globally, these traits must be designed for deployment across a wide range of germplasm, agronomic management, and environments. In this section, we unpack what is currently known about the traits underpinning the CERCA ideotype, explore their molecular and physiological foundations, and discuss avenues for engineering and deploying these traits through advanced breeding and genetic engineering approaches. We begin by examining cold tolerance followed by a focus on recycling nitrogen, highlighting opportunities to keep nitrogen on farm.

### Cold tolerance to extend the season

Earlier planting and faster spring growth allow for better use of available light, water, and natural nitrogen by tapping into the spring nitrogen cycle, reducing nitrogen losses that typically occur when bare soil is exposed. This involves planting earlier in the spring and creating maize with increased growth rates under low temperatures (Fig. 1B). Currently, “…maize is a cold-sensitive plant whose physiological reactions to sub-optimal temperatures are well understood, but their molecular foundations are only beginning to be deciphered” (Sowiński et al. 2020). While some maize germplasm is chilling tolerant (Yi et al. 2021), maize is largely unable to survive freezing events after the meristem has emerged from the soil (Greaves 1996; Nielsen 2020). Across angiosperms, cold-related traits, such as the ability to experience cold acclimation, vernalization, and endodormancy, have evolved multiple times, separately, but often through similar genetic pathways (Preston and Sandve 2013). Maize may also have these pathways, and thus, the ability to survive cold if induced properly. The Andropogoneae tribe, to which maize belongs (Welker et al. 2020), includes both annual and perennial species, such as *Tripsacum dactyloides* and *Miscanthus*, that have independently evolved cold and frost tolerance (Gallaher et al. 2022). The presence of frost-tolerant relatives suggests that engineering frost tolerance in maize may be feasible.

The goals for earlier maize planting are: germination at 6 °C, growth at 10 °C, and frost tolerance to −2 °C. Generally, temperature below 15 °C is considered suboptimal and inhibits growth (Greaves 1996). Temperatures below 13 °C create difficulties developing a functional photosynthetic apparatus (Hardacre and Eagles 1980) that manifests in 50% reduction in photosynthesis (Rotundo et al. 2019). Despite these challenges, young maize seedlings exhibit significant phenotypic plasticity for sustained growth at suboptimal temperatures (Riva-Roveda et al. 2016). Moreover, modern phenotyping approaches make it easier to select for photosynthetic resilience (and therefore, growth at low temperatures), enabling the breeding approaches required to develop early-season germplasm (Fracheboud et al. 1999).

#### The physiology of cold tolerance

Plants experience chilling and freezing temperatures as 2 distinct stresses. As such, physiological challenges require distinct solutions. Hallmarks of a general chilling response are osmolyte accumulation, membrane lipid composition changes, and hormone alteration (Graham and Patterson 1982). There is also a reliance upon calcium signaling to prevent dehydration-related damage as temperatures warm and cells rehydrate (Ding et al. 2019; Ghosh et al. 2021; Shomo et al. 2024). Freezing poses additional challenges, including damage to cells caused by ice crystallization. Many freezing-tolerant plants harness supercooling mechanisms to prevent freezing and also induce distinct lipid changes (Bredow et al. 2018; Ritonga and Chen 2020; Barnes et al. 2023). Due to ice nucleation in the apoplast, freezing can induce large changes in water potential (Jeffree et al. 1987), which means drought tolerance and chilling/freezing tolerance pathways have many elements in common (Medeiros and Pockman 2011). In some cases, they are thought to have evolved from common mechanisms (Das et al. 2023), although debate exists on the exact evolutionary relationships between the 2 in grasses (Stolsmo et al. 2024).

**Figure 4.**
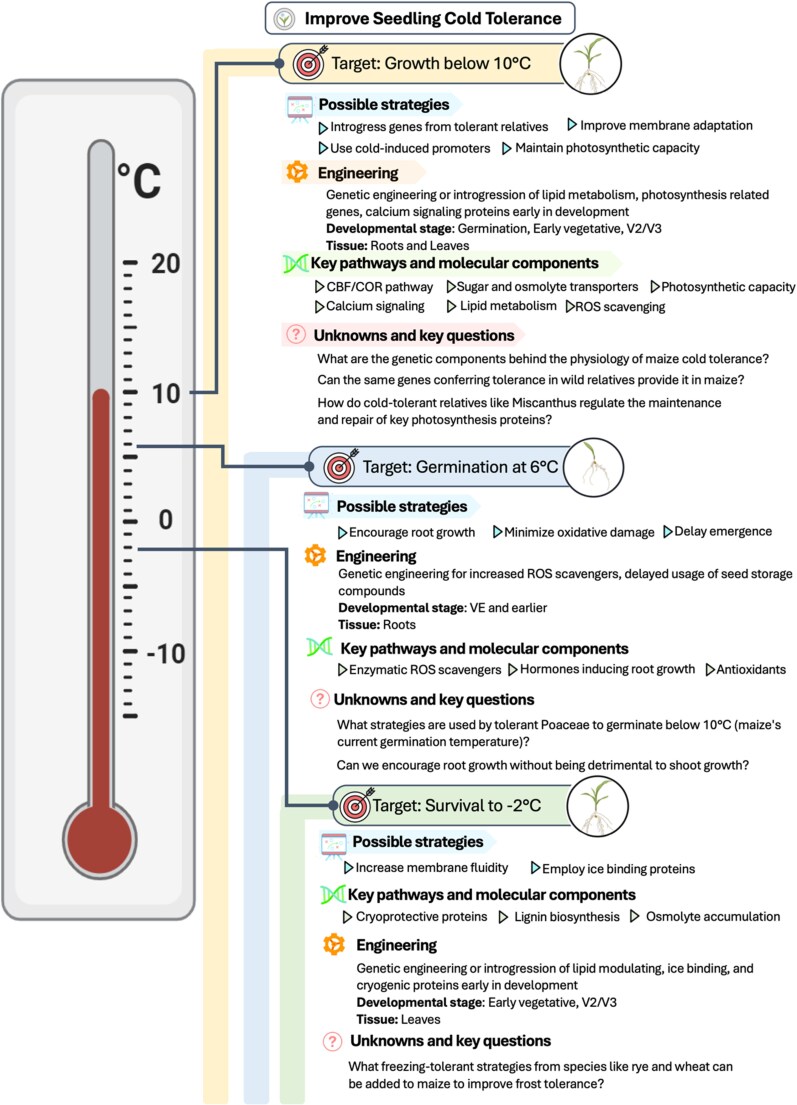
Cold tolerance to tap into the spring nitrogen cycle and extend the photosynthetic window. ROS, Reactive Oxygen Species. Developmental stages (VE, Vegetative Emergence; V, Vegetative [V1-V3]) are based on Abendroth et al. (2011).

To grow in suboptimal chilling conditions, maize must germinate at lower temperatures, and transition to autotrophic growth (Fig. 4). Both traits are highly responsive to temperature, and decades of study have led to documented genetic diversity for germination and early seedling growth (Miedema 1987; Greaves 1996). Not surprisingly, lines that more rapidly mobilize seed reserves often emerge faster under cold (Eagles 1982). Recent work in rice has shown that roots, and their biochemical traits, play a key role in seedling establishment during chilling stress (Hsu and Hsu 2019). Maize varieties exhibiting a higher root / shoot growth ratio under low temperatures at seedling emergence tend to be more tolerant to cold (De Fenza et al. 2017).

After transitioning to autotrophic growth, maize experiencing chilling temperatures must photosynthesize to continue surviving. Both chloroplast biogenesis and photosynthetic capacity suffer heightened sensitivity to cold (Pimentel et al. 2005; Rotundo et al. 2019). However, some cold-tolerant maize lines can maintain the synthesis of chlorophyl, anthocyanin, and carotenoids along with undisrupted chloroplast development in the cold (Grzybowski et al. 2019). Maize's photosynthetic capacity significantly decreases when grown in chilling temperatures (Long and Spence 2013; Rotundo et al. 2019). The reduction of photosynthesis in all plants is most severe in the presence of both high light and chilling temperatures. In chilling temperatures in the dark, photosynthesis has been shown to decrease and anthocyanin production to increase (Szalai et al. 1996). In the light, the decrease in carbon assimilation is exacerbated by an increase in photoinhibition and damage to the D1 protein of photosystem II (PSII; Öquist 1983; Long 1983). PSII is not properly degraded and replaced, leading to overexcitation. The oxygen-evolving side of PSII has been shown to be one of the most sensitive components of the photosynthetic apparatus in many plants, including beans and cucumbers (Long 1983; Öquist 1983). By decreasing photoinhibition and increasing CO_2_ assimilation in cold temperatures, we can improve the photosynthetic capacity and growth of maize.

#### The genetic basis of cold tolerance

Conventional plant breeding has improved maize cold tolerance in its earliest growth stages in temperate climates (Grzybowski et al. 2019). Numerous studies demonstrate the current existence of natural cold-tolerant variations within certain maize germplasm (Hund et al. 2008; Revilla et al. 2014, 2016; Yi et al. 2021; Zeng et al. 2021; Jiang et al. 2022; Li et al. 2022b), and its relation to root traits (Hund et al. 2008) and grain yield (*r* = 0.48; Mock and McNeill 1979). These natural variations present a valuable opportunity to pinpoint key alleles, genes, and pathways that are associated with seedling cold tolerance by comparing genomics, transcriptomics, proteomics, and phenomics in diverse maize germplasm collections. Well-characterized, low-temperature related proteins are obvious targets to explore. Some examples of this include lipid metabolism genes like fatty acid desaturases and HPC1 (Ariizumi et al. 2002; Sakamoto et al. 2004; Barnes et al. 2022), sugar and osmolyte accumulation genes like TPP1 and P5CS (Hur et al. 2004; Pramanik and Imai 2005), signaling components like CNGC (Cui et al. 2020; Wang et al. 2021), and the well-known CBF/COR/ICE pathway (Hwarari et al. 2022).

However, in order to make significant progress in the development of temperature resilient maize, cross-species molecular genetics and genomic analyses must also be examined (Li et al. 2016; Sowiński et al. 2020; Zhang et al. 2020; Zhou et al. 2022b). Studying low-temperature tolerant grasses and maize relatives is the first step in identifying genes and processes to improve germination and establishment. Small grains such as spring wheat, barley, oat, and rye can be sown much earlier in the spring, in part because of their cold tolerance during germination and early growth (Collier et al. 2020). Modeling other species’ transcriptional responses via machine learning technologies can enhance our understanding of how maize can be genetically engineered for improved cold tolerance (Meng et al. 2021; Zhou et al. 2022a). Cross-species analyses can unveil conserved or convergent cold-tolerance genes and pathways that are missing, invariant, or inactive in maize, as well as illuminate proteins of interest to compare structure and function-based differences.

Comparative genetic and genomic approaches have been particularly successful in understanding the basis of photosynthetic efficiency in cold temperatures. *Miscanthus* can quickly degrade and replace PSII D1 protein at chilling temperatures, maintaining photosynthesis (Spence et al. 2014). In the same study, *Miscanthus* increased the amount of the photoprotective pigment zeaxanthin, diverting excess light energy away from the photosynthetic apparatus, whereas maize did not. Other comparative studies of maize and *Miscanthus* (Naidu et al. 2003; Naidu and Long 2004; Wang et al. 2008; Spence et al. 2014) identified 2 enzymes, Rubisco and pyruvate orthophosphate dikinase, thought to be limiting photosynthesis at low temperatures. These make promising targets for genetic engineering. It is important to note however, that most of what we currently know in this area is based on a single or a few maize cultivars that are not representative of the enormous genetic diversity within the species. Similarly, diverse *Miscanthus* cultivars may react differently than what has previously been studied.

#### Engineering cold tolerance

The first step in discovering potentially useful genetic variation will be to screen germplasm from ecologically relevant geographies in the targeted selection environment. This approach was highly successful in identifying populations of *Miscanthus* with superior chilling tolerance (Głowacka et al. 2015). Populations of maize introgression lines, diverse germplasm, such as heirloom varieties, and maize wild relatives can all be employed at this stage. Selection of germplasm for further analysis will be done by simulating a range of low-temperature germination scenarios with a soil-based thermal gradient table (Welbaum et al. 2016). From there, new target genes must be identified and can be added to those listed previously as known players in cold tolerance. For example, the abundance of key proteins, such as those involved in D1 repair and in the light-independent reactions, could be increased to improve the photosynthetic capacity of maize in the cold.

### Optimizing nitrogen use: recycling and stabilization on the farm

**Figure 5.**
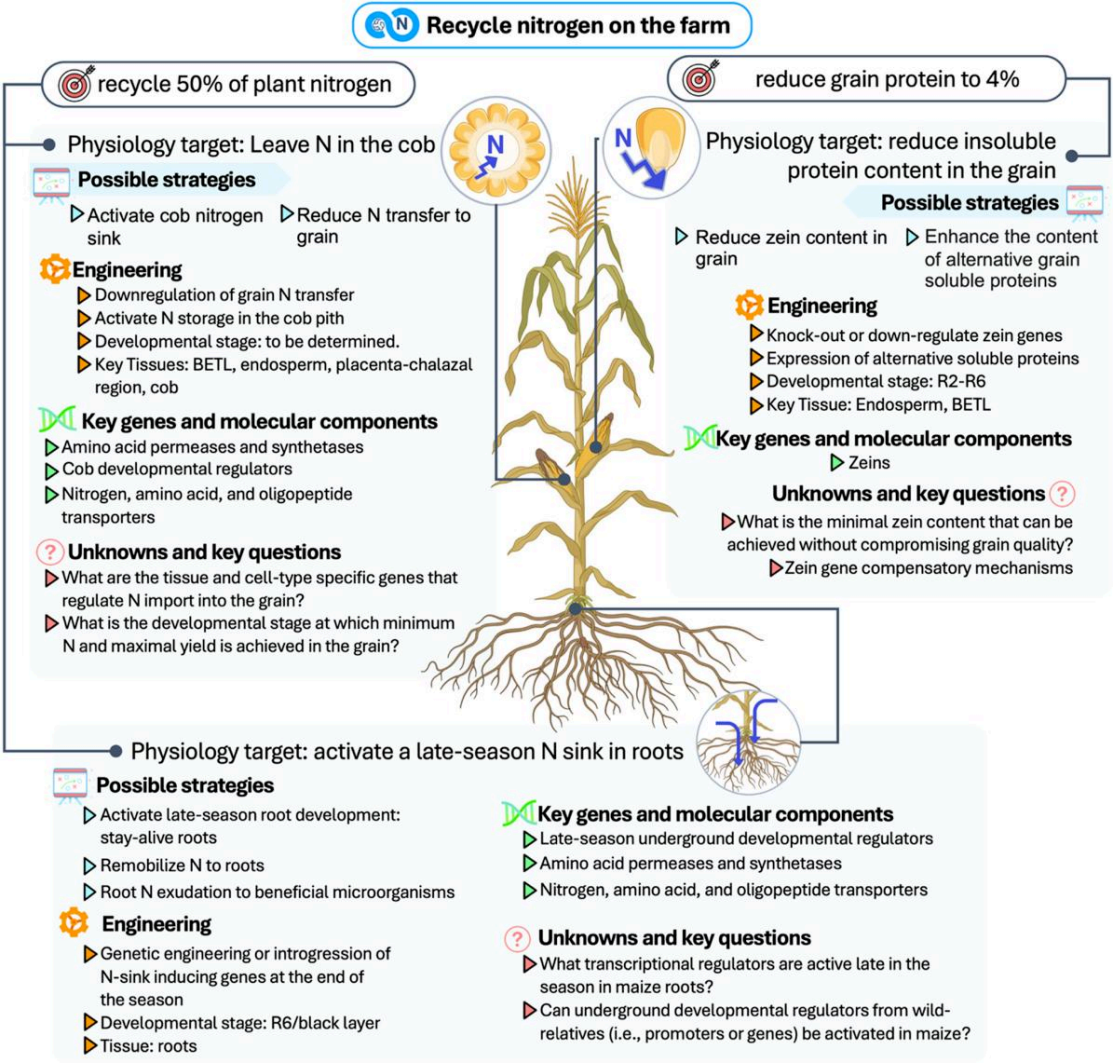
Strategies and targets to recycle nitrogen on the farm. Developmental stages (V, Vegetative [V1-V10]; R, Reproductive [R1-R6]; R6 is also known as black layer stage) are based on Abendroth et al. (2011). BETL, Basal Endosperm Transfer Layer.

**Figure 6.**
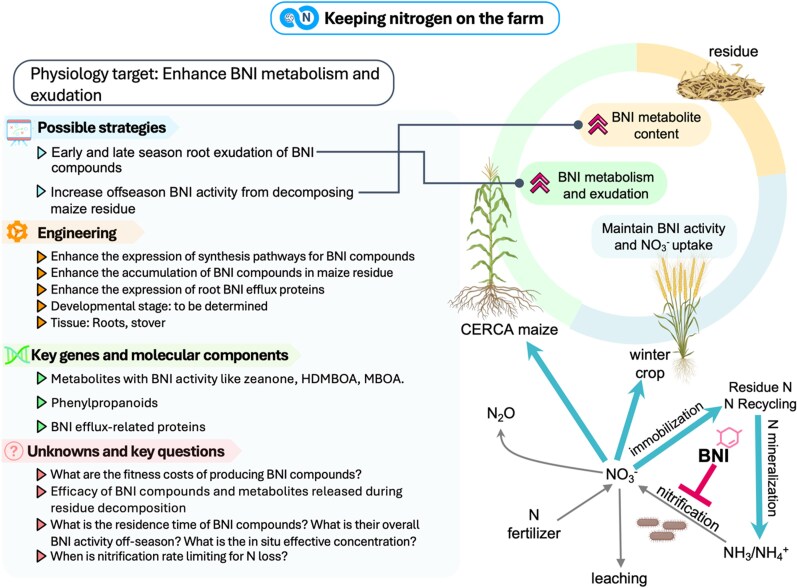
Keep nitrogen on the farm with BNI. HDMBOA (2-hydroxy-4,7-dimethoxy-1,4-bezoxazin-3-one); MBOA (6-methoxy-2-benzoxazolinone).

The strategies for cold tolerance outlined above address the challenge of synchronizing maize growth with nitrogen availability during the early spring. The CERCA approach aims to enhance nitrogen efficiency on farms while improving yield by extending the growing season. Complementing these efforts, reducing the nitrogen demand of the grain and activating alternative nitrogen sinks after grain filling has been completed could enable the recycling of nitrogen on farm (Fig. 5). Enhanced BNI could offer the opportunity to keep nitrogen on the farm by stabilizing soil nitrogen throughout fall, winter, and early spring. As illustrated in Fig. 6, BNI targets the microbial processes responsible for converting ammonium to nitrate, reducing nitrogen. In the following, we outline potential strategies to engineer these traits in maize.

#### Reduce low-quality storage proteins in the grain

Protein content is approximately 8% to 10% in commercial yellow dent varieties, translating to ∼1.5% grain nitrogen content (Loy and Lundy 2019). Our goal is to reduce grain protein content to 4% without impacting plant growth and yield. This corresponds to ∼0.75% grain nitrogen, which we refer to as minimum grain nitrogen in subsequent sections. Indeed, selection for yield resulted in significant reductions in grain nitrogen content (Mueller et al. 2019a) and likely underpin genetic gain in growth and yield due to the favorable energy balance. The grain's protein is mainly stored in the endosperm (Wu et al. 2022), where zeins comprise up to 60% of the protein content and serve as the main nitrogen sink (Larkins 2019). Zeins are insoluble, hydrophobic proteins with a poor amino acid balance, which make them low-quality proteins for many downstream industrial processes and livestock feed (Calvez et al. 2021). Thus, reducing zein content is key to diminishing grain nitrogen demand (Fig. 5). Our strategy for reaching minimum grain nitrogen focuses mainly on ∼80% zein reduction to lower grain protein content. Enhancing the content of alternative soluble proteins (Kriz and Wallace 1991) to decrease zein content, is an alternative to balance the amino acid content in the grain, but only if it can be achieved without affecting yield and grain storability.

It is important to distinguish our low grain nitrogen objective from Quality Protein Maize (QPM) breeding. QPM was developed to enhance grain nutritional quality by increasing lysine and tryptophan content and reducing zein accumulation (Maqbool et al. 2021). Although both QPM and CERCA target zein downregulation, our goals differ: QPM enhances protein quality, while CERCA seeks to reduce total nitrogen investment in the grain, by lowering overall protein content, to improve NUE on farms (Fig. 3). CERCA's nitrogen aim is high-quality–lower quantity, while QPM's nitrogen aim is high-quality–high quantity. Modeling and selection suggest there are fundamental plant physiological tradeoffs with yield and fertilizer demand that limit QPM (Maqbool et al. 2021), while CERCA is avoiding this tradeoff through an integrated livestock feed-cropping systems approach.

QPM lines rely on *opaque2* mutant alleles that alter the protein profile of the grain and result in reduced zein content (Hasjim et al. 2009). The endosperm-specific opaque2 (O2) family of transcription factors regulates the accumulation of both zein and nonzein (soluble) proteins in the grain (Schmidt et al. 1992; Zhang et al. 2015b). In *opaque2* mutants, decreased zein levels are accompanied by increased expression of soluble proteins such as globulins, leading to elevated concentrations of essential amino acids like lysine and tryptophan (Mertz et al. 1964). However, these nutritional gains come with agronomic tradeoffs: *opaque2* mutants show reduced yields, delayed kernel drying, and heightened susceptibility to disease and pests (Maqbool et al. 2021). These limitations are likely due to O2's broader regulatory roles including its control over genes involved in carbon and nitrogen metabolism (Li et al. 2015). Recent findings by Yang et al. (2024) provide mechanistic insights into O2-dependent starch accumulation in the kernel and its broader role in sink strength, highlighting the complexity of its role in kernel development. Together, these findings underscore the need for a more targeted approach to zein reduction and clarify why *opaque2* mutations are not suitable tools to achieve low-protein grain.

#### Leave nitrogen in the cob after minimum grain nitrogen is achieved

Maize cobs were shown to decompose by approximately 33.5% after 150 days in soil due to their high fiber content in one of the few studies to investigate this (Zou et al. 2021); however, a wide range in decomposition is expected based on temperature, moisture, microbial communities, and tillage practices (Burgess et al. 2002). Nevertheless, these studies highlight the potential of the cob for the slow return of nitrogen back to the soil during the following growing season (Fig. 5). During the early stages of ear development, the cob pith actively metabolizes amino acids to set up nitrogen transfer to the kernel (Seebauer et al. 2004). While downregulating amino acid synthesis and translocation from cob to grain represents an interesting prospect to reduce grain nitrogen demand, it is prudent to investigate how this intervention affects the rate of ear growth and nitrogen accumulation in the cob and grain due to their relationship with final kernel weight and yield (Mueller et al. 2019b). Key enzymes to achieve this goal include those that regulate the balance of amino acids and their translocation to the cob (Seebauer et al. 2004; Pan et al. 2015). Activating the expression of storage proteins in the cob pith tissue could contribute to the generation of a competing nitrogen sink that would reduce amino acid transport to the grain.

#### Activate a late-season nitrogen sink in roots

Like the cob, roots could provide an alternative nitrogen sink to recycle nitrogen post-harvest. Due to their higher lignin content and lower concentration of water-soluble carbon and nitrogen, root tissues decompose more slowly than leaves and are less susceptible to volatilization or runoff because they are embedded in the soil (Rasse et al. 2005; Sun et al. 2018). Being a tissue with slow decomposition, roots could provide an organic structural reservoir for gradual nitrogen release in soil. This holds true for grasses, in which root residue consistently decomposes more slowly than leaf residue, and residues from perennial species decompose more slowly than those of annual species like maize (Shi et al. 2013). In maize, root residues contribute at least 10 times less to nitrification and to nitrous oxide emissions than when root and leaf residues are mixed (Rummel et al. 2020), suggesting that redirecting nitrogen to roots may enhance in-field retention and reduce environmental losses.

The goal is to remobilize nitrogen to roots late in the season in a manner that does not affect yield or plant performance, i.e. after physiological maturity of the grain (black layer formation). This strategy comes from perennial species that remobilize nitrogen in their above-ground biomass and transport it to below-ground tissues to overwinter (Dohleman et al. 2012; Yang et al. 2016; Mitros et al. 2020). One main strategy would be to activate root growth late in the season. As an analogy to stay-green, we propose “stay-alive” roots that would provide a developmental sink for nitrogen (Fig. 5). The molecular mechanisms activated during plant senescence to facilitate nitrogen remobilization from leaves also offer promising prospects for remobilizing nitrogen (Havé et al. 2017). Another interesting possibility would be to activate root nitrogen efflux directly to beneficial microorganisms, like arbuscular mycorrhizal fungi and plant-growth promoting rhizobacteria (Yuan et al. 2018; Ramírez-Flores et al. 2020). Further research is required to determine the optimal balance between efficiency, crop yield, microbial interactions, and environmental suitability.

#### Engineering nitrogen recycling

The stay-green trait present in maize and sorghum varieties provides a route to both keep the nitrogen on the field and enhance yield, as photosynthesis is maintained at high level until grain fill is completed (Kamal et al. 2019; Zhang et al. 2019). The deployment of alternative nitrogen sinks and low protein grain in stay-green maize varieties could work synergistically to keep nitrogen on the field and increase carbohydrate yield in grain. While selection led to genotypes with increased capacity for nitrogen uptake and retention in leaves (Mueller et al. 2019a; Fernandez et al. 2022), deploying nitrogen recycling traits, particularly those seeking to remobilize nitrogen to roots, will require genetic engineering and/or introgression from maize wild relatives.

The discovery and use of genes and promoters that are active in key tissues and cell-types is required to engineer nitrogen recycling without yield expense. For example, the identification of promoters that are active in the basal endosperm transfer layer is key, because of its role in transporting metabolites from the maternal tissue to the endosperm (Sabelli and Larkins 2009). The placenta-chalazal region (Wu et al. 2022) and other cob tissues involved in the regulation of nitrogen transfer to the kernel are also of high importance. Research will also be needed to identify promoters with root-specific activity late in the season. Late-season promoters might not work to lower nitrogen grain demand as reducing nitrogen translocation may be necessary once minimum grain nitrogen has been achieved (i.e. before black layer). In the case of the downregulation of targets, gene editing and genetic engineering tools may be deployed to knock-out or knock-down targets in specific tissues. The latter is a useful approach to avoid knocking out essential enzymes for nitrogen metabolism outside of the spatiotemporal target.

#### Possible challenges and open questions

The engineering of nitrogen recycling poses challenges and questions. Reducing grain protein content by downregulating or knocking out zeins might compromise grain durability and yield. This is because zeins are essential for the formation of endoplasmic reticulum-derived protein bodies, which contribute to the hard vitreous texture of the kernel (Holding and Larkins 2008). Disrupting zein synthesis can lead to a soft, opaque endosperm, making kernels more susceptible to mechanical damage and pest infestation (Hasjim et al. 2009; Guo et al. 2013). Knocking out specific zein genes may trigger compensatory upregulation of other zein or nonzein proteins (Guo et al. 2013). Data suggest that the lower biological limit for protein content in maize is 5% (Woore et al. 2021). A century of selection for low protein in the Illinois Long-Term Selection Experiment plateaued at around 5% grain protein with the lowest reported value being 3.71%, which would correspond to ∼0.7% grain nitrogen content (Moose et al. 2004; Dudley and Lambert 2010), slightly below our target of 0.75%. The minimal zein content that can be achieved without compromising kernel quality will have to be determined. An additional possible strategy for engineering this or other traits would take advantage of the hybrid nature of maize production and place complementary alleles in each heterotic group. The inbred parents and their hybrid seed would be normal, but the hybrid plants and grain (what farmers harvest) would express the complemented trait as designed.

Figuring out nitrogen storage strategies and the dynamics of source-to-sink relationships is needed to develop alternative nitrogen sinks. Annual species, including maize, do not develop specialized storage organs (Hjertaas et al. 2022), weakening the sink strength potential for underground nitrogen remobilization. The mechanisms controlling the relative sink strength of roots and seeds, a key difference between annual and perennial plants are currently unknown (Lundgren and Des Marais 2020). Recent identification of molecular components that regulate the establishment of physiological and developmental sinks in perennial grasses (Yang et al. 2016; Mitros et al. 2020) offers promising insights to remobilize nitrogen.

#### Can we keep nitrogen on farm by boosting BNI?

The reduction of nitrogen loss through BNI presents many avenues to increase the sustainability and productivity of agroecosystems (Fig. 6). Current advances in BNI research have focused on, among other strategies, root exudation of compounds with BNI (Subbarao et al. 2013; Coskun et al. 2017). The recent identification of maize BNI compounds like zeanone, 2-hydroxy-4,7-dimethoxy-1,4-bezoxazin-3-one (HDMBOA) and 6-methoxy-2-benzoxazolinone (MBOA; Otaka et al. 2022, 2023), supports the notion that exudation of compounds with BNI activity is a potential strategy to keep nitrogen in soil. While promising, the potential of BNI to reduce nitrogen losses in annual cropping systems remains uncertain (Subbarao et al. 2021; Hartman et al. 2022).

Synthetic nitrification inhibition (SNI), however, can provide an upper-bound benchmark for BNI's impact. Meta-analyses show SNI can increase NUE by an average of 17% (Burzaco et al. 2014), while reducing nitrogen leaching and N_2_O emissions by up to 50% (Lam et al. 2022). Collectively, SNIs and other enhanced-efficiency fertilizers could contribute to global nitrogen savings of approximately 1.25 Tg yr^−1^ (Wang et al. 2024). Nonetheless, BNI may impose a metabolic cost that could reduce plant nitrogen uptake, potentially limiting its net benefit (Kuppe and Postma 2024). The effectiveness of nitrification inhibition, biological or synthetic, is highly context-dependent, shaped by interactions among environment, soil type, and agronomic practices (Woodward et al. 2021; Beeckman et al. 2024).

Modeling based on field studies suggests modest gains in nitrogen retention caused by BNI during the growing season (Hartman et al. 2022). In contrast, the nongrowing season represents a greater opportunity for retention, as 30% to 50% of annual nitrogen inputs are lost during this period. Therefore, it is yet to be determined whether root exudation of BNI compounds early in the season can inhibit nitrification at field scale. Increased BNI activity in root exudates combined with earlier planting enabled by cold tolerance (Fig. 6) could help maximize the potential to reduce nitrogen loss. In addition to root exudates, increased end of season BNI activity (Fig. 6) could lower nitrogen losses in fall and early spring and retain the potential rise in soil nitrogen inputs in the fall caused by recycling nitrogen to the roots and cob (Fig. 5).

Here, we propose modifying the biochemical composition of maize residue, the above and belowground biomass left after grain harvest, to extend BNI activity into the off-season. Currently an underexplored strategy, the potential of residue to slowly release BNI compounds provides an opportunity for continual BNI activity during its degradation in the fall and spring. While the accumulation of soluble BNI metabolites during senescence holds promise for producing residue with BNI capacity, achieving long-term BNI activity depends on the persistence of these compounds and/or their gradual release during residue degradation. Lignin has the slowest rate of degradation in soil compared with other carbohydrates in residue (Templeton et al. 2009; Thapa et al. 2023), making it a compelling candidate as a carrier for slow release of BNI metabolites. Furthermore, lignin and BNI compounds are linked, as both are synthesized through the phenylpropanoid pathway and the breakdown of lignin produces BNI compounds (Nardi et al. 2013; Issifu et al. 2024). A possible drawback of BNI residue is that changes in lignin composition could result in lower grain yield (Sattler et al. 2010) or increased disease susceptibility (Kolkman et al. 2023). Genotypes with extreme lignin composition can be used to elucidate the effect of residue composition on soil nitrogen loss (Barrière et al. 2004; Takeda et al. 2018). How the variation in lignin composition could alter nitrification and other nitrogen cycling traits in different soil types and environments remains an open question. While further research into all these approaches is needed, we believe that the combination of in-season and off-season nitrification inhibition, including the use of cover crops with enhanced BNI activity, is a promising strategy to reduce nitrogen losses in the field. Continued research is needed to assess BNI's impact and how this trait could be integrated into current agricultural systems.

### Unleashing genetic diversity to extend the season and reduce fertilizer inputs in corn production

Over millennia, millions of farmers, scientists, plant breeders, and engineers have contributed to the development of the current agricultural production system. However, opportunities for system-wide (re)design have been rare. Today, breakthroughs in comparative genomics, phenomics, artificial intelligence (AI), and multiscale modeling offer unprecedented avenues to address key challenges in maize agriculture, such as improving cold tolerance, increasing nitrogen efficiency, and enabling longer photosynthetic windows. Genetic diversity serves as the foundation for achieving the CERCA ideotype, as it could allow scientists to unlock traits that have yet to be fully optimized for overcoming these barriers. Over the past decades, genetic mapping and allele mining in maize have proven remarkably effective, especially when paired with genomic selection to integrate thousands of relevant loci (Lorenz et al. 2011; Crossa et al. 2017). However, modern breeding has already extensively exploited the available diversity for key traits such as chilling tolerance and nitrogen efficiency. To achieve the next leap in maize production, maize must tap into a broader pool of genetic diversity beyond conventional breeding lines.

Fortunately, there are various germplasm resources to explore: landraces, wild *Zea* relatives, *Tripsacum*, and the broader C_4_ tribe Andropogoneae. Recent advances in genetic analysis of traditional varieties have enabled high-resolution characterization of thousands of maize accessions, combining genomic data, environmental profiling, and field evaluations. These resources are now being leveraged to identify traits such as cold germination, chilling tolerance, and variation in grain nitrogen content. For instance, studies like those by Revilla et al. (2014) and Barnes et al. (2022) have demonstrated the potential of diversity derived from traditional varieties for these traits. Early maturing and cold-tolerant Northern European Flint materials have been instrumental in extending maize cultivation to temperate regions and offer valuable genomic resources for improving cold tolerance in elite germplasm (Haberer et al. 2020). Transcriptomic analyses have identified genes and regulatory mechanisms under cold stress, cold tolerance and growth under cold conditions (Frey et al. 2020). The value of these materials is further underscored by the identification of alleles that enhance early-stage photosynthesis under cold conditions (Urzinger et al. 2024), using a double haploid library generated from European Flint landraces (Mayer et al. 2020). These findings illustrate the value of locally adapted landraces to broaden the genetic diversity of elite germplasm.

Beyond cultivated varieties, wild relatives of maize also harbor genetic variation, particularly for traits such as cold tolerance and perenniality. Among the wild relatives of maize, *Z. mays* ssp. *mexicana* stand out as sources of additional variation for cold tolerance (Barnes et al. 2022). However, due to extensive historical introgression between maize and *Z. mays ssp. mexicana*, much of this diversity may already be present in maize germplasm (Yang et al. 2023). *Z. diploperennis*, a perennial relative, offers significant genetic potential for nutrient remobilization (Westerbergh and Doebley 2004; Ma et al. 2019), although its adaptive cues seem more suited to highland tropical environments, which may limit its utility. Valuable resources such as TeoNAM and NIL populations have been developed (Chen et al. 2019; Guerrero-Corona et al. 2021), providing powerful tools to study and transfer useful traits from these relatives into maize.

*Tripsacum dactyloides*, commonly known as eastern gamagrass, only diverged from maize about 600,000 years ago (Chen et al. 2022). As a native grass of the US Corn Belt, *Tripsacum* exhibits unique traits, including seedling frost tolerance down to −6 to −8 °C (deWet et al. 1982; Yan et al. 2019). Temperate accessions from *T. dactyloides* demonstrate nitrogen remobilization during the fall (Heggenstaller et al. 2009; Kiniry et al. 2024), aligning well with the goals of the CERCA maize ideotype. Using diverse *Tripsacum* accessions collected across the US, CERCA researchers have developed F_1_ and F_2_ populations that display variation in these critical traits. These populations enable genetic and expression QTL mapping, aiding in the discovery of key regulatory genes involved in cold tolerance and nutrient recycling.

Beyond the genus *Zea*, the broader Andropogoneae tribe offers vast untapped genetic potential. Advances in grass genomics have provided new opportunities for studying these species, which include both grain crops and perennial biofuel and turfgrass species. Research groups within our initiative have assembled genome sequences for nearly 800 species and varieties of grasses, enabling mapping efforts for traits such as perenniality and environmental adaptation. Additionally, we are conducting physiological, RNA, and protein profiling experiments in several dozen wild species under both field and controlled conditions. These studies are calibrating genomic models and identifying the critical genes and regulatory patterns needed to achieve the CERCA ideotype. By tapping into these diverse germplasm resources, we can expand the genetic toolkit available for maize breeding, enabling innovative solutions to challenges in cold tolerance, nitrogen efficiency, and economically and environmentally improved sustainable agricultural production.

Recent advancements in multiscale modeling, phenomics, AI, and genetic engineering are further revolutionizing maize improvement. Crop modeling tools like APSIM (Holzworth et al. 2014) and DSSAT (Hoogemboom et al. 2019) integrate genetic and environmental data to predict plant performance and optimize breeding strategies (Messina et al. 2022; Cooper and Messina 2023). High-throughput phenotyping, including remote sensing with drones and satellites, is improving trait selection for cold tolerance and senescence (DeSalvio et al. 2022; Li et al. 2022a; Adak et al. 2023; Alahmad et al. 2024). AI models trained on DNA and protein data (Jumper et al. 2021; Elnaggar et al. 2022; Zhai et al. 2025), are accelerating the discovery of novel genes and regulatory elements, enabling more precise trait development. These advances, combined with cutting-edge genetic engineering techniques such as modular cloning (Engler et al. 2014) and developmental gene facilitated transformation (McFarland et al. 2023), are streamlining the introduction of beneficial traits into elite maize germplasm. These innovations, combined with comparative genomics and the study of maize's wild relatives, are enabling the CERCA initiative to reimagine corn production by improving nitrogen efficiency and climate resilience.

## Concluding remarks

Ensuring sustainable nutrient management in corn production is essential for creating an agricultural system that supports both yield and ecological health. The CERCA approach to temperature and nitrogen can complement, and enhance, other advancements in carbon fixation and phosphorus efficiency and contribute to a more balanced and resilient agricultural system that minimizes environmental losses and maximizes resource utilization. These advancements provide building blocks for designing traits that can be modularly introduced into breeding programs. By allowing the temperate industrialized agriculture industry to integrate these traits incrementally and strategically, this approach ensures compatibility with larger breeding efforts, paving the way for sustainable and high-performing maize varieties.

The CERCA initiative is focused on advancing sustainable and resilient corn agriculture by solving maize's growing challenges in temperate environments. Cold tolerance should enable the extension of the growing season, increase yields, and expand the light interception period while aligning plant nitrogen uptake with the soil's nitrogen cycle. This synchronization should help reduce nitrogen loss, lower environmental pollution, and decrease nitrogen fertilizer expenses for farmers. CERCA aims to solidify corn's role at the heart of a bioeconomy that prioritizes efficient nitrogen use, resilience, and reduced ecological impact. By leveraging current advances in genetic engineering, AI-driven analysis, and comparative genomics, maize production can be transformed to benefit farmers through higher yields and reduced nitrogen costs, while also mitigating environmental leakage of nitrogen. The time to act is now.

## Data Availability

No new data were generated or analysed in support of this research.

## Dive Curated Terms

The following phenotypic, genotypic, and functional terms are of significance to the work described in this paper:

cold tolerance Planteome: TO:0000303
